# RAS/RAF/MEK/ERK and PI3K/PTEN/AKT Signaling in Malignant Melanoma Progression and Therapy

**DOI:** 10.1155/2012/354191

**Published:** 2011-10-12

**Authors:** Ichiro Yajima, Mayuko Y. Kumasaka, Nguyen Dinh Thang, Yuji Goto, Kozue Takeda, Osamu Yamanoshita, Machiko Iida, Nobutaka Ohgami, Haruka Tamura, Yoshiyuki Kawamoto, Masashi Kato

**Affiliations:** Unit of Environmental Health Sciences, Department of Biomedical Sciences, College of Life and Health Sciences, Chubu University, Kasugai, Aichi 487-8501, Japan

## Abstract

Cutaneous malignant melanoma is one of the most serious skin cancers and is highly invasive and markedly resistant to conventional therapy. Melanomagenesis is initially triggered by environmental agents including ultraviolet (UV), which induces genetic/epigenetic alterations in the chromosomes of melanocytes. In human melanomas, the RAS/RAF/MEK/ERK (MAPK) and the PI3K/PTEN/AKT (AKT) signaling pathways are two major signaling pathways and are constitutively activated through genetic alterations. Mutations of RAF, RAS, and PTEN contribute to antiapoptosis, abnormal proliferation, angiogenesis, and invasion for melanoma development and progression. To find better approaches to therapies for patients, understanding these MAPK and AKT signaling mechanisms of melanoma development and progression is important. Here, we review MAPK and AKT signaling networks associated with melanoma development and progression.

## 

Cell signaling pathways are important for understanding not only cancer progression but also all life phenomena, including regulation of cell growth and death, migration, and angiogenesis [1–4]. Moreover, the events are accurately controlled by various intracellular signal transduction molecules [2, 5–7]. In cancer progression, the signaling is hyperactivated and/or silenced irreversibly. These irreversible losses of control in signal transduction allow cancers to acquire cancer-progression-specific phenotypes, such as antiapoptosis, abnormal proliferation, angiogenesis, and invasion. Previous studies revealed that collapse of signaling control was induced by both genetic and environmental factors [8–12].

Melanin-producing cells, acquired in several species from fungi to primates in the long evolutionary process, have many advantageous functions for survival strategy [13–19]. Melanocytes, melanin-producing cells that are the origin of melanoma, are developed from neural crest cells with several types of cell signaling pathways and gene expression [15, 20–22]. Human melanomas are categorized as nevus-associated melanomas and de novo melanomas based on their developmental process. Nevus-associated melanomas are transformants of preexisting benign lesions, and their malignant conversion progresses in a multistep manner [23–26]. De novo melanomas develop without pre-existing benign lesions [6, 27–29]. In humans, most melanomas are thought to have developed de novo. RFP-RET transgenic mice of line 304/B6 (RET mice) are powerful tools for analyses of melanoma with pre-existing benign lesions [6, 30, 31]. The entire process of melanoma development via tumor-free, benign, premalignant, and malignant stages in RET mice corresponds to the multistep melanomagenesis in humans [32]. Recently, we identified ZFP 28, CD109, and c-RET as melanoma-related molecules through analysis of tumors in RET mice [4, 33, 34]. 

Melanoma progression is closely associated with oncogenic change: (1) genetic alteration (heritable changes in the DNA sequence such as gene mutations, deletions, amplifications, or translocations) and (2) epigenetic alteration (modulated transcriptional activities by DNA methylations and/or by chromatin alterations). Much information associated with melanoma development such as information on gene mutations, alterations of gene expression patterns, and protein activities has been reported.

The RAS/RAF/MEK/ERK pathway, one of the most well-known pathways involved in melanoma progression, is regulated by receptor tyrosine kinases, cytokines, and heterotrimeric G-protein-coupled receptors [35]. The small G protein RAS (HRAS, KRAS, and NRAS in humans) is localized to the plasma membrane and activates a downstream factor, RAF (ARAF, BRAF and CRAF in humans) followed by sequential activation of MEK and ERK, and this signal is finally transduced to regulation of transcription in the nucleus (Figure 1) [36]. This pathway is constitutively activated by growth factors such as stem cell factor (SCF), fibroblast growth factor (FGF), hepatocyte growth factor (HGF), and glial-cell-derived neurotrophic factor (GDNF) [37, 38], though activation of this signal is weak in melanocytes.

ERK is hyperactivated in 90% of human melanomas [39] by growth factors [40] and by genetic alterations of upstream factors, RAS, and RAF proteins [41]. In humans, *NRAS *and* BRAF* genes are mutated in 15% to 30% and in 50% to 70% of human melanomas, respectively, leading to their permanent activation [41] followed by promotion of proliferation, survival, invasion, and angiogenesis of melanoma [42, 43]. BRAF signaling is also associated with NF*κ*B promoter activity. Inhibition of BRAF signaling decreased NF*κ*B promoter activity associated with survival, invasiveness and angiogenesis for melanoma formation [44, 45].

PTEN, containing a phosphatase domain, is inactivated in 12% of melanomas through mutation or methylation [46]. A substrate of PTEN, phosphatidylinositol (3,4,5)-trisphosphate (PIP_3_) and phosphorylates AKT [47], which activates cell survival, proliferation, cancer promotion, and antiapoptotic signaling through mTOR (mammalian target of rapamycin) and NF-*κ*B pathways in melanoma (Figure 1) [48–51]. RAS can also bind and activate PI3K, resulting in increased AKT activity [52]. MDM2 is a ubiquitin ligase that targets p53 (an apoptosis-associated tumor-suppressor protein) for degradation and is highly expressed in 6% of dysplastic nevi, 27% of melanoma in situ, and 56% of invasive primary and metastatic melanomas [53]. MDM2 is also a substrate for AKT [54–56]. Taken these results indicate that AKT/MDM2 pathway is involved in melanoma progression (Figure 1). 

 Recently, many persistent studies developed therapeutics and drugs for melanomas. Phase 2 study for melanoma patients was tested by using the combination of bevacizumab, an inhibitor of angiogenesis, and everolimus, an inhibitor of mTOR which is a downstream target of PI3K/PTEN/AKT signaling. In this study, 12% of malignant melanoma patients achieved major responses [57]. Plexxikon (PLX4032) is a novel selective inhibitor for BRAF^V600E^, a major activated mutation observed in 60% of human melanomas [41]. This inhibitor is dramatically effective in 74–80% of patients with BRAF^V600E^-positive melanomas [58–60]. However, tumors grow and progress again in almost all patients from about 7 months after initial treatment of PLX4032 [58, 60]. Recent studies have revealed that treatment with PLX4032 activates a novel pathway leading to regrowth and reprogression of tumors with bypass of BRAF signaling, resulting in tumors acquiring resistance to the BRAF inhibitor [61–65]. Molecular-based targeted treatments are usually effective only in a subset of patients, and predictive molecular tests are required to identify tumors with an activated targeted pathway and to select patients with a good chance of response. On the other hand, treatment with bortezomib, a NF-*κ*B inhibitor, alone or combined with paclitaxel and carboplatin showed no clinical effect on malignant melanoma patients in phase 2 study even though NF-*κ*B is a downstream target of RAF and AKT [66, 67]. These limited effects indicate that signaling pathways in malignant melanomas may compensate each other to make resistance to molecular-targeted therapy. Thus, molecular mechanisms of melanoma development and progression are complicated and melanoma therapy is still incomplete. Further studies and a better understanding of melanoma development and progression are needed to establish effective therapeutics with few harmful side effects.

## Figures and Tables

**Figure 1:**
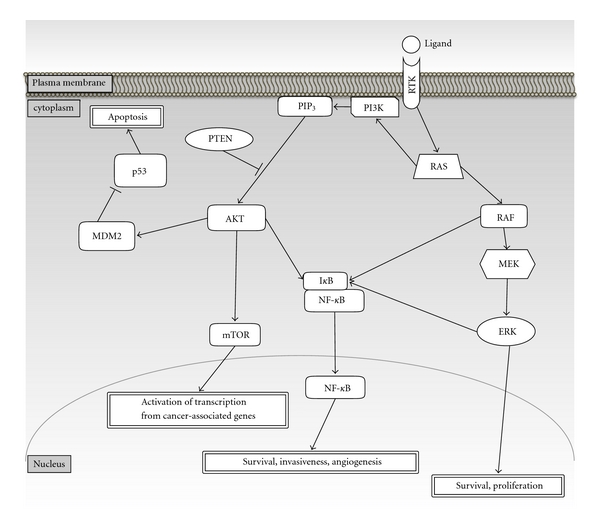
Signal transduction in melanoma development and progression. Extracellular signaling (ligand) triggers intracellular signaling through receptors such as tyrosine kinases (RTK). Triggered signals are transduced via verious factors, including tyrosine kinases, phosphatases, inhibitors, cofactors, and transcription factors and affect melanoma development and progression. Abbreviations: AKT thymoma viral proto-oncogene; MDM2 transformed mouse 3T3 cell double minute 2; mTOR mechanistic target of rapamycin; PI3K Phosphoinositide 3-kinase, PIP_3_, Phosphatidylinositol (3, 4, 5)-trisphosphate; PTEN phosphatase and tensin homolog.
